# Methods for scaling up an outreach intervention to increase colorectal cancer screening rates in rural areas

**DOI:** 10.1186/s43058-023-00540-1

**Published:** 2024-01-08

**Authors:** Jennifer Coury, Gloria Coronado, Jessica J. Currier, Erin S. Kenzie, Amanda F. Petrik, Brittany Badicke, Emily Myers, Melinda M. Davis

**Affiliations:** 1 Oregon Rural Practice-Based Research Network, Oregon Health & Science University (OHSU), 3181 SW Sam Jackson Park Rd, Portland, OR 97239 USA; 2 Kaiser Permanente Center for Health Research, Portland, USA; 3 Division of Oncological Sciences, Knight Cancer Institute, OHSU, Portland, USA; 4 OHSU-PSU School of Public Health, OHSU, Portland, USA; 5 Department of Family Medicine, OHSU, Portland, USA

**Keywords:** Colorectal cancer screening, Mailed fecal immunochemical testing, Patient navigation, Scaling-up interventions, Implementation science, Rural health, Multilevel intervention

## Abstract

**Background:**

Mailed fecal immunochemical test (FIT) outreach and patient navigation are evidence-based practices shown to improve rates of colorectal cancer (CRC) and follow-up in various settings, yet these programs have not been broadly adopted by health systems and organizations that serve diverse populations. Reasons for low adoption rates are multifactorial, and little research explores approaches for scaling up a complex, multi-level CRC screening outreach intervention to advance equity in rural settings.

**Methods:**

SMARTER CRC, a National Cancer Institute Cancer Moonshot project, is a cluster-randomized controlled trial of a mailed FIT and patient navigation program involving 3 Medicaid health plans and 28 rural primary care practices in Oregon and Idaho followed by a national scale-up trial. The SMARTER CRC intervention combines mailed FIT outreach supported by clinics, health plans, and vendors and patient navigation for colonoscopy following an abnormal FIT result. We applied the framework from Perez and colleagues to identify the intervention’s components (including functions and forms) and scale-up dissemination strategies and worked with a national advisory board to support scale-up to additional organizations. The team is recruiting health plans, primary care clinics, and regional and national organizations in the USA that serve a rural population. To teach organizations about the intervention, activities include Extension for Community Healthcare Outcomes (ECHO) tele-mentoring learning collaboratives, a facilitation guide and other materials, a patient navigation workshop, webinars, and individualized technical assistance. Our primary outcome is program adoption (by component), measured 6 months after participation in an ECHO learning collaborative. We also assess engagement and adaptations (implemented and desired) to learn how the multicomponent intervention might be modified to best support broad scale-up.

**Discussion:**

Findings may inform approaches for adapting and scaling evidence-based approaches to promote CRC screening participation in underserved populations and settings.

**Trial registration:**

Registered at ClinicalTrials.gov (NCT04890054) and at the NCI’s Clinical Trials Reporting Program (CTRP no.: NCI-2021–01032) on May 11, 2021.

Contributions to the literature

- This research describes a way to scale up a complex colorectal cancer screening outreach in organizations that want to improve cancer screening rates in rural populations. Scaling up means that the approach is flexible enough to work in different types of organizations that either provide health care to people or partner with healthcare providers.
- We describe how we teach organizations about mailing fecal tests to their populations and navigating patients to colonoscopies.
- We describe how we will measure if we succeed in sharing the approach with these organizations so others can learn from our study methods.

## Background

Colorectal cancer (CRC) is the third largest cause of cancer deaths in the USA despite the availability of highly effective screening that can reduce mortality and morbidity [1–3]. Mailed fecal immunochemical test (FIT) outreach and patient navigation are evidence-based practices shown to improve rates of CRC screening in various settings. Multiple systematic reviews and meta-analysis of mailed FIT outreach have reported average improvements in CRC screening of 22–28 percentage points [4–8]; this approach also reduced disparities in CRC outcomes between Black and non-Hispanic White populations in a large integrated system [9]. Patient navigation, in which a trained individual assesses patient barriers and delivers tailored educational and emotional support, can further address barriers to screening [7, 10–12]. A recent meta-analysis of patient navigation programs for CRC screening showed a 64% relative improvement over usual care [13]. Nevertheless, neither mailed FIT outreach nor patient navigation have been broadly adopted by health systems and organizations that serve rural and Medicaid populations.

The reasons for low adoption are multi-factorial. Some health systems have limited information technology resources or infrastructure to identify patients who are eligible for CRC screening and track key outcomes [14, 15]. Limited electronic health record (EHR) data can cause difficulties in identifying and targeting patient populations for interventions [16–18]. Another key barrier in many under-resourced health systems is a lack of adequate staffing. High staff or leadership turnover can lead to restructuring the organization and many additional hours for training new staff [16, 19, 20]. Another common barrier is the time required for staff to implement a centralized outreach program, particularly using patient navigation as part of it [21–23]. For a program like the centralized mailed FIT program described above, organizations relied on existing staff with various roles to implement the program components, causing extra time burden with other competing work priorities [16, 24]. Furthermore, while ultimately cost-effective [25, 26], patient navigation and mailed FIT require initial financial investments and can thus be perceived as costly and unsustainable in resource-limited settings [21, 22]. Budget impacts range from US $25.50 more per patient for FIT completion navigation compared to usual care [27] to US $275 or more per patient for navigation through completion of all screening [25]. For complex, multi-level interventions such as these, approaches are needed to introduce both the scientific evidence and implementation strategies to organizations in a way that encourages adoption.

A pragmatic trial tested the implementation, effectiveness, and maintenance of a mailed FIT test and patient navigation program to improve rates of CRC screening and follow-up in clinical practices serving rural Medicaid enrollees [10]. While spreading an evidence-based practice such as this one involves replicating that practice in additional similar organizations, we want to examine ways to “scale up” a multi-level intervention to different types of organizations and addressing system issues that arise during full-scale implementation [28–30]. Here, we describe the protocol for a scale-up study that is rolling out a mailed FIT and patient navigation intervention to rural organizations and studying effective approaches for scaling up a complex, multi-level intervention. We describe how we identified the intervention’s core functions [31] and present the design for the evaluation of scale-up activities.

## Methods

This paper describes the scale-up protocol of the SMARTER CRC project, a two-phase study that includes a pragmatic trial followed by a scale-up trial. SMARTER CRC is being conducted as part of the National Cancer Institute-funded Accelerating Colorectal Cancer Screening and Follow-up through Implementation Science (ACCSIS) consortium [32]. The overall aim of ACCSIS is to conduct multi-site, coordinated, transdisciplinary research to evaluate and improve CRC screening processes using implementation science strategies. The protocol for the main trial has been previously reported [10]. Briefly, the first phase of SMARTER CRC is a large-scale, cluster-randomized trial involving 3 health plans and 28 rural clinics serving populations with Medicaid insurance. The trial tests an intervention to improve rates of CRC screening using a collaborative model involving health plans, direct-mail vendors, and affiliated clinics to deliver mailed FIT outreach [33] and patient navigation for follow-up colonoscopy [34]. We refer to that here as the “SMARTER CRC intervention” and consider it to be a complex, multicomponent health intervention as defined by Perez Jolles and colleagues [31]. The mailed FIT outreach component relies on best practices identified during the CDC-sponsored mailed FIT summit [5], and the patient navigation component uses an adapted version of the phone-based New Hampshire Colorectal Cancer Screening Program [35]. Implementation was supported by training (i.e., 5-h multi-model patient navigation training; 1-h training on how to review patient lists) and practice facilitation delivered by members of the study team.

Both phases in SMARTER CRC involved a collaborative partnership between the Oregon Health & Science University’s (OHSU) Oregon Rural Practice-based Research Network and the Kaiser Permanente Northwest Center for Health Research. A regional advisory board of local practitioners, patient representatives, CRC, and public health experts met quarterly and informed the pragmatic trial; a national advisory board of CRC experts, rural health experts, patient organization representatives, and national public health experts meets quarterly and is informing the scale-up trial. SMARTER CRC has obtained approval from the OHSU’s Institutional Review Board (protocol number: 20681), which has granted a waiver of informed consent. A ceding agreement was obtained from the Kaiser Permanente Center for Health Research.

### Tailoring implementation strategies for scale-up

The goals for phase 2 of the SMARTER CRC study is to refine the SMARTER CRC intervention for additional types of organizations and test it in the scale-up portion of the study which is as follows:

- Partner with regional and national organizations (n ~ 20) to scale up the program to clinics serving rural and underserved patients in high-priority geographic regions of the US (n ~ 130 clinics; 17,000+ patients) using collaborative learning, workshops and webinars, and practice facilitation. Assess training delivered, program adoption and adaptations, and determinants of dissemination success.

Findings from the cluster-randomized trial were used to inform modifications to the SMARTER CRC intervention to prepare for scale-up [36–38]. Qualitative findings from analysis of clinic contact logs, interviews with clinics and health plans, and periodic reflections with practice facilitators were synthesized and made available to ECHO faculty to inform development of the ECHO didactic sessions. Findings pertained to strategies for designing CRC outreach programs (e.g., including providers in planning and mailing in batches for large populations), tailoring outreach to patient populations (e.g., developing culturally sensitive materials), identifying patients for FIT mailing (e.g., filtering out inactive patients), increasing patient uptake (e.g., using messages from providers), and reducing clinic-level implementation barriers (e.g., developing patient navigation guides). These strategies were identified based on actual or desired adaptations during the cluster-randomized study [39]. To inform scale-up planning, we used causal-loop diagramming, a method from systems science, to identify causal mechanisms underlying the SMARTER CRC multi-level intervention, as understood by the study team [40]. Second, using these qualitative research data, we also identified factors associated with implementation success [39].

Based on these findings and conversations with the national advisory board, we identified three objectives for our scale-up activities: (1) focus on CRC screening outreach for rural communities, (2) teach a diverse set of organizations multicomponent and multi-level approaches to raise CRC screening and follow-up rates, and (3) provide tools for organizational change and implementation strategies to support the outreach intervention.

### Scale-up methods and strategy

In our pragmatic trial, the SMARTER CRC intervention leverages partnerships between clinics, health plans, vendors, and the study team to share the burden of implementation. Table 1 presents the intervention broken down into *intervention components*, or major categories of the SMARTER CRC intervention along with the *determinants*, which are contextual factors driving the need for each component. For each component, present the *function* or purpose of the activities, the *form* the activities take as modified from the initial pragmatic trial, and the s*cale-up dissemination approach* to how those components are shared with recruited organizations [28, 31, 41].

**Table 1:** SMARTER CRC intervention functions, forms, and scale-up approach

Intervention components	Function	Form (intervention activities)	Scale-up dissemination approach
Planning	Build motivation for CRC screening outreach and organizational investment and buy-in	1. Provide scientific rationale and making the case for this model, including cost-effectiveness evidence 2. Identify an organizational champion 3. Conduct an organizational self-assessment	ECHO series presentations: Building a business case, choosing an implementation model Facilitation guide
Workflow assessment	Support consistent implementation by developing a workflow collaboratively to better inform staffing and choose the best approach for all organizations	1. Teach the basics of mailed FIT programs, patient navigation to colonoscopy 2. Hold meeting with implementing organizations to develop a workflow for mailed FIT and patient navigation 3. Create a workflow document and validate it with participants	ECHO series presentation: Build engagement and implement a Collaborative Cancer Screening Program Workshop Facilitation guide & sample workflows
Learning collaborative meetings	Create a learning collaborative environment, share additional training Troubleshoot implementation as roll-out happens across multiple organizations	1. Hold monthly meetings to bring all outreach partners together 2. Share implementation data monthly regarding mailed FIT and navigation outreach	Facilitation guide & toolkit
FIT selection	Ensure organizations are selecting high-quality FITs to ensure ability to distribute FITs by mail, high patient response, and accurate abnormal test rates	1. Share expert information about FIT quality and types and assist with FIT selection 2. Coach participants on how to choose distribution option a. Variable FIT b. Centralized FIT	ECHO series presentation: Build engagement and implement a Collaborative Cancer Screening Program Facilitation guide Technical assistance for organizations regarding FIT choices
Eligible patient identification	Support and provide tools to identify patients to ensure that patients who are truly eligible receive FITs Ensure clinics were up to date on mailed FIT eligibility guidelines for their patients	1. Present different models of generating eligible patient list either generated by health plans based on claims and validate the list using EHR data or generated by other organizations 2. Present and share “scrub” training that teaches CRC screening eligibility guidelines	ECHO series presentation: Generating the eligible patient list Facilitation guide with scrub training templates Technical assistance Report templates
Patient prompts and reminders	Use primers, such as texts, phone calls, and printed mailings before mailed outreach to increase return rates Use reminders to initial non-completers to increase the return rate per best practice	1. Send introduction letters, phone calls, or text messages before the FIT mailing 2. Send reminders—mailed, phone, text, email, text-video after the FIT mailing to nonresponders or whole population	ECHO session: Patient outreach and communication Letter and text templates and scripts Videos Patient engagement webinar
Mailing FITs	Remove the burden of mailing from clinical practices by using vendors, health plan support Address challenges with generic FIT instructions and literacy	1. Mail FITs to everyone deemed eligible; include postage or a return USPS business reply code on the mailers to increase return 2. Use vendors for the mailing (optional) 3. Use a brief, easy to read invitation letter, plus simple FIT completion instructions customized per organization 4. Completed FITs are mailed back to either a lab or a clinic (or dropped off by patient)	ECHO session: Designing a mailed FIT program Technical assistance Outside resources
Patient navigation	Help increase staff support to transition patients from primary care to specialty care (i.e., for colonoscopy) where there is a severe gap in patient support	1. Teach the importance of patient navigation and why it is needed 2. Identify and train patient navigators at the clinic level or at the health plan level in navigation to follow-up colonoscopy	ECHO session: Navigating patients to follow-up colonoscopy Facilitation guide Patient navigation training class

We worked with our national advisory board to develop a plan to scale up the program and disseminate training to a national audience. The first part of this work involved defining which functions of the intervention would be shared with new organizations. Then the scale-up plan was developed and included learning collaboratives informed by the Extension for Community Healthcare Outcomes (ECHO) project [42], virtual workshops and webinars, technical assistance, and a supporting facilitation guide. ECHO-informed learning collaboratives were chosen to facilitate participation by busy rural and remote healthcare organizations. Because ECHO supports case-based learning, we opted to supplement the ECHO learning collaboratives with workshops and webinars to build specific content-area expertise. In addition, the research team also used the Translational Science Roadmap to Impact to establish audience for recruitment, topics for dissemination, and components that were core elements of the implementation support [43].

### Recruitment

Our study’s scale-up recruitment targets clinical practices and hospital systems, community organizations, health departments, health plans (national or regional), and tribal clinic systems. To better serve contexts likely to be encountered during scale-up, we have expanded the types of organizations from those enrolled in the pragmatic trial (i.e., Oregon Medicaid health plans and clinical practices) to a wider array of organizational types and explore how the intervention could flex to new environments. To be eligible for participation in the scale-up, organizations must have interest in improving rates of CRC screening and/or follow-up in the populations they serve and commit to attending the ECHO sessions and completing data collection activities. A list of potential participants for scale-up outreach was developed that includes organizations unable to take part in the initial pragmatic trial but interested in the topic, recommended contacts from advisory board members and their networks, settings with prior relationships with study team members, and ECHO series registration requests. Additional leads were gathered using a snowball sampling approach, warm-handoff referrals, or by partners contacting the research team directly after hearing regional or national presentations.

The study team (principal investigators, project managers, practice facilitators) works in partnership with local, regional, and national research partners to recruit representatives from these organizations. The study team developed recruitment materials, including an email template, a recruitment flyer, and a slide presentation which describe the scale-up program and include a link to register for the ECHO series. In addition, the Oregon ECHO Network—which hosts our ECHO scale-up activities—regularly publishes information about upcoming opportunities, including the CRC Outreach ECHO description. The team tracks outreach using an in-house clinic relationship management tool built in Microsoft Access; this tracking included reasons for participation or declining to participate.

### Scale-up dissemination approach

To spread the SMARTER CRC program to the recruited organizations, we take a multipronged approach to sharing program components and materials. Implementation research has shown that dissemination of a toolkit alone is not sufficient to spark organizational change [44]. The scale-up dissemination activities include collaborative learning, a facilitation guide and other materials, individualized technical assistance, a patient navigation workshop, and webinars (Table 1).

#### Collaborative learning (ECHO)

ECHO is a collaborative learning program that uses tele-mentoring to support systems to improve health care quality. During ECHO sessions, healthcare providers and other participants use telecommunication technology to deliver and receive training, education, and support that build team capacity. A typical ECHO session consists of a 15-min expert presentation followed by a case study led by an attendee (such as a clinic staff or organizational representative). For SMARTER CRC, we designed a six-session ECHO series, with topics focused on the following: (1) building the business case for CRC outreach, (2) building engagement, (3) designing a mailed FIT program, (4) identifying patients who are due for CRC screening, (5) delivering patient communications (FIT and reminders), and (6) navigating to follow-up colonoscopy. The series is delivered twice between March and November 2023.

A facilitation guide and a set of local and national resources on mailed FIT and patient navigation, compiled by the research team, is provided to ECHO attendees. Following the ECHO series, participants are invited to attend a workshop on patient navigation, training webinars on multi-level communications and implementation challenges, and are eligible for organization-specific technical assistance in any of the topics covered during the ECHO series.

#### Patient navigation workshop

The multimodal patient navigation training program developed for the main trial is offered to ECHO attendees to support asynchronous and synchronous interactive learning. The 5-h workshop addresses the importance of CRC screening and follow-up, barriers to CRC screening, and the patient navigation protocol. An optional pre-recorded video on motivational interviewing is also available for attendees.

#### Training webinars

Participants in the ECHO and patient navigation workshop are invited to participate in 1-h webinars based on organizational need and desired skill development. The webinars build content-specific knowledge and share best practices. The topics of the webinars include workflow design, common implementation challenges, patient engagement, and tailoring outreach to underserved patient populations.

#### Technical assistance

Consistent with the pragmatic trial, scale-up practice facilitation activities are primarily facilitated by the project manager, practice facilitators, and other study team members with relevant expertise. During the ECHO series, participating organizations are offered a chance to sign up for a 1-h technical assistance session. These technical assistance meetings address the key goals and objectives identified by the participating organization, and team members provide expertise in the relevant area.

### Data collection

Scale-up evaluation includes summative surveys, interviews following the ECHO, and formative satisfaction surveys following each session as described in detail below.

#### Baseline, post-ECHO, and 6-month follow-up surveys

At the launch of the ECHO learning collaboratives, workshops, and webinars, registered participants are asked to complete a brief (15-min) survey. Baseline survey questions assess current clinical practices related to CRC screening and follow-up (e.g., current CRC screening practices and policies, screening test use, and organizational resources). Additional questions are based on the Consolidated Framework for Implementation Research (CFIR) [45, 46] and relate to organizational readiness, internal context (leadership, staffing, resources, culture/teamwork, communication, organizational capacity), and external context (geography, policy). We use adapted questions from prior ECHO assessment tools to assess organizational learning processes, organizational knowledge creation, and training content. For all participants, we gather demographic information (e.g., age, sex), professional role, affiliated organization name and location, and email address. Participants of multiple SMARTER CRC training events are asked to complete a single baseline survey.

After the last ECHO session, another brief survey (20 min) is distributed to participants covering satisfaction with the ECHO, what they learned during the ECHO, clinical practices related to CRC screening, and intentions to adopt the program components. We use questions adapted from the Program Sustainability Assessment Tool (PSAT) [47] to assess organizational knowledge transfer, organizational knowledge retention, and workflow integration. Questions about capacity for sustainability, also adapted from the PSAT, include organizational support, funding stability, partnerships, and organizational capacity.

Six months following the administration of the baseline survey, study staff email respondents an invitation to complete a follow-up survey. This survey asks respondents about their clinical practices related to CRC screening and follow-up, the extent to which implementation of the SMARTER CRC program is being planned or executed, planned or implemented adaptations to the program, and their participation in SMARTER CRC trainings or workshops.

#### Satisfaction surveys

Brief satisfaction surveys are administered immediately following each ECHO learning collaborative session (six sessions delivered twice) and following each workshop or webinar. The satisfaction survey gathers participant demographics, role, and affiliation and asks about their overall satisfaction with the session, areas for improvement, and remaining questions they have.

#### Qualitative interviews

At least one member of each organization participating in the ECHO series is invited to take part in a semi-structured qualitative interview conducted about 3 months after completion of the last ECHO session. Interviews seek to understand contextual factors, barriers, and facilitators to implementation and sustainment, ECHO program acceptability, adaptations (both desired and executed), and unanticipated consequences (positive or negative). Interviews are conducted via videoconference and generally last 30–60 min. All qualitative interviews will be digitally recorded, professionally transcribed, uploaded to ATLAS.ti, and then analyzed by the research team using an immersion crystallization approach to identify salient themes [48].

### Scale-up evaluation

The scale-up evaluation is focused on recruited organizations and has three objectives: (1) assess engagement in scale-up activities, (2) determine implementation of a mailed FIT test and patient navigation program as a result of engagement in scale-up activities, and (3) describe adaptations of core components of a CRC screening outreach program and the rationale for modifications (Table 2).

**Table 2:** Measures and data sources for SMARTER CRC scale-up

Measure	Sub-measures	Data sources	Timeline				Framework
			Baseline	Mid-ECHO	ECHO endpoint	6-month post ECHO	
**Engagement** Organizational-level participation; characteristics of participating organizations/staff; participant satisfaction with scale-up activities	N organizations/staff who attend (1) ECHO learning collaborative series (by session), (2) patient navigation workshop, (3) post-ECHO webinars; N organizations/staff who request technical assistance	Registration & attendance for ECHO learning collaborative series	√	√			RE-AIM; CSAT
		Patient navigation workshop and webinars			√		
		Technical assistance requests		√	√		
	Participant objectives and satisfaction with the following: (1) the ECHO learning collaborative series, (2) patient navigation workshop, (3) post-ECHO webinars	Baseline ECHO series survey	√				
		ECHO series endpoint survey			√		
		Post patient navigation workshop survey			√		
		Post-webinar survey			√		
	Topics of participants’ questions during ECHO learning collaborative series, patient navigation workshop, post-ECHO webinars, and technical assistance	ECHO session observation notes	√	√			
		Technical assistance meeting notes		√	√		
		Key-informant interviews				√	
**Adoption** Number, proportion, representativeness of settings/staff who implement program, by program component: (1) mailed FIT test and (2) patient navigation; reasons for adopting program	N, type of organization (health plan, clinic, other) and staff (care coordinator, navigator) that implement the program, by core component (“form”) (mailed FIT, patient navigation) or peripheral component to improve CRC screening, follow-up or referral to care (“function”)	6-month post-ECHO survey				√	RE-AIM; PSAT
	Reason(s) for adopting program, by component; implementation strategies; program sustainability	6-month post-ECHO survey & key-informant interviews				√	
**Adaptation** How organizations modified CRC program components: (1) what program component or components were modified, (2) nature of the modification(s), and (3) reasons/rationale modification(s) were made	N, nature of program modifications, by program component	6-month post-ECHO survey				√	CSAT; PSAT; FRAME; Going to Full Scale
		Key-informant interviews				√	
	Reason(s) modifications were made, by modification; implementation strategies; program sustainability	Key-informant interviews				√	

#### Engagement

Engagement is defined as organizational behavior (as measured by participation) and affect (as measured by self-reported satisfaction) with scale-up activities [49]. We will compare engagement within and across recruited organizations to assess how scale-up strategies lead to adoption of any CRC screening components. Organizational engagement is measured through multiple data sources. We use attendance logs to track the number of individuals and organizational representatives who attend our ECHO sessions, patient navigation workshop, webinars, and individualized technical assistance sessions. Research staff observe training and collaborative learning sessions to qualitatively assess levels of engagement by organizations. We maintain study materials on the ECHO website and track downloads of facilitation guide and templates. We collect the number of individuals (by role) and organizations (by type) who participated in each collaborative learning, training, and technical assistance session. These data will be analyzed using quantitative and qualitative methods.

#### Adoption

Adoption measures the number, proportion, and representativeness a mailed FIT and patient navigation program components implemented by recruited organizations (per the RE-AIM framework). Using the post-ECHO survey and 6-month follow-up survey data, we assess the number of organizations that have facilitated the delivery of the SMARTER CRC intervention (i.e., worked with another organization to implement program components) and the number of health plans or clinics who have begun to implement each component of the intervention (i.e., mailed FIT and patient navigation) and characterize adoption similarities and differences across recruited organization. We assess degree of implementation and fidelity with which program components are implemented using the RE-AIM framework [50–52].

#### Adaptations

Using FRAME-IS (i.e., the Framework for Reporting Adaptations and Modifications to Evidence-based Implementation Strategies), adaptations are defined as modifications made to a mailed FIT and patient navigation program components and an organization’s rationale for these changes [53]. Among organizations who have implemented a mailed FIT or patient navigation program, we collect and report information about adaptations to the program and reasons adaptations were made. We use data from 6-month follow-up surveys, key informant interviews, and meeting notes from technical assistance sessions to track adaptations to the program and implementation support. We classify adaptations using components of the FRAME (Framework for Reporting Adaptations and Modifications-Enhanced) for intervention adaptations and the FRAME-IS framework for implementation strategy adaptations [54, 55], focusing on adaptation goal, type, and the reason the adaptation was made. We build on prior application of the FRAME framework by our team to track factors such as who decided to make an adaptation and whether it was made proactively or reactively in response to an identified need [56].

#### Sustainability

Sustainability is defined as the extent to which a mailed FIT and patient navigation program are institutionalized in organizational practices and policies (RE-AIM framework). Using 6-month follow-up survey data and findings from key informant interviews, we assess the potential for sustainability across the clinics or organizations that implement each component of the program (i.e., mailed FIT and patient navigation). We assess potential for sustainment using PSAT domains (i.e., funding stability, partnerships, organizational capacity, program evaluation, communications, and strategic planning). We measure the extent to which program components become part of the routine organizational practices and policies using the RE-AIM framework [52].

## Discussion

The SMARTER CRC scale-up study assesses a multicomponent mailed FIT and patient navigation intervention scaled up to be delivered to many types of organizations that serve rural populations. For the SMARTER CRC intervention to become sustainable without the research team support provided in the pragmatic trial, primary program implementation needs to shift from research-led to organization-led. During the scale-up study, we are able to assess the adaptations made by adopting organizations in order to implement a complex multi-level intervention. Our findings may impact how to broadly scale evidence-based CRC screening programs in rural settings.

The Translational Science Roadmap to Impact from the Translational Science Benefits Model [43] enables us to establish the core ECHO learning collaborative foci and recruitment targets. This model includes measurable indicators of clinical and community health impacts when examining translation of evidence-based practices into community interventions and clinical applications. The Going to Full-Scale framework [28] is another model for informing the potential adoption mechanisms and implementation support systems needed to broadly scale a program. The steps in the Barker et al. framework include setting up the program through planning and partner engagement, developing a “scalable unit” (i.e., the more generalizable SMARTER CRC intervention), testing the intervention in contexts likely to be encountered at full scale, and going to full scale (which facilitates adoption by a larger number of sites). Consistent with the framework, we can evaluate adoption mechanisms (e.g., engaging champions, training staff) and support systems (e.g., tracking systems) needed to achieve scale-up.

We chose to use the core components of the intervention implemented in the pragmatic trial but to leave the actual forms of those components flexible for the implementing scale-up organizations. We did this by presenting a facilitation guide that could be used in a flexible manner, a learning collaborative where organizations learned from each other in addition to expert faculty presenters, and individualized sessions where the material could be customized to different settings. We used the qualitative interview findings from the pragmatic trial and our advisory board input to indicate areas where the intervention needed refinement as we scaled up to new and different types of organizations. Planned interviews with organizations that adopt the intervention may further identity strategies to facilitate the implementation in practice.

While several existing frameworks [29, 30, 41, 45, 57] address scaling up evidence-based practices, some frameworks do not easily translate to specific situations. While the Barker et al. framework [28] gave us some insight into how to describe our work, ultimately we had to ask additional questions to apply this framework to our intervention and settings. We also could consider the Aarons et al. (2017) concept of “scaling out” evidence-based interventions (EBIs) to either new populations or delivery systems. Applying this model, we would say we both are scaling up, which they define as when an EBI designed for one setting is expanded to other health delivery units that are the same or very similar, and scaling out, with variants of implementation to different populations. In our case, some participating organizations serve populations that differ from the populations served by the clinical practices in the pragmatic trial. This scale-out framework is flexible enough to account for changes from the pragmatic trial to the spread to additional organizations.

Literature about scaling up multi-level interventions is evolving. Figuring out the suitable type and intensity of implementation strategies to support scale-up is also evolving. Our evaluation intends to inform the selection of core components for a CRC screening outreach program, whether the dissemination strategies we chose lead to successful adoption of any of the components and implementation strategies to scale a complex, multi-component intervention for rural healthcare settings.

## Data Availability

Not applicable.

## Abbreviations

ACCSIS: Accelerating Colorectal Cancer Screening and Follow-up through Implementation Science
CRC: Colorectal cancer
CFIR: Consolidated Framework for Implementation Research
PSAT: Program Sustainability Assessment Tool
CSAT: Clinical Sustainability Assessment Tool
ECHO: Extension for Community Healthcare Outcomes
FIT: Fecal immunochemical test
OHSU: Oregon Health & Science University
FRAME: Framework for Reporting Adaptations and Modifications–Expanded
FRAME-IS: Framework for Reporting Adaptations and Modifications to Evidence-based Implementation Strategies
