# Correction: Methods for scaling up an outreach intervention to increase colorectal cancer screening rates in rural areas

**DOI:** 10.1186/s43058-024-00550-7

**Published:** 2024-01-24

**Authors:** Jennifer Coury, Gloria Coronado, Jessica J. Currier, Erin S. Kenzie, Amanda F. Petrik, Brittany Badicke, Emily Myers, Melinda M. Davis

**Affiliations:** 1 Oregon Rural Practice-Based Research Network, Oregon Health & Science University (OHSU), 3181 SW Sam Jackson Park Rd, Portland, OR 97239 USA; 2 Kaiser Permanente Center for Health Research, Portland, USA; 3 Division of Oncological Sciences, Knight Cancer Institute, OHSU, Portland, USA; 4 OHSUPSU School of Public Health, OHSU, Portland, USA; 5 Department of Family Medicine, OHSU, Portland, USA

**Correction: Implement Sci Commun 5, 6 (2024)**

**https://doi.org/10.1186/s43058-023-00540-1**

Following the publication of the original article [1], the authors reported an error in the Competing Interest statement which reads “Dr. Coronado serves as a member of the Board of Scientifc Advisors for the National Cancer Institute (NCI). The rest of the authors declare they have no competing interests.”

The correct Competing Interest statement should have read “From 2020- 2022, Dr. Coronado served as a scientific advisor for Exact Sciences, through a contract with the Kaiser Permanente Center for Health Research. From 2021 – 2023, Dr. Coronado served as Principal Investigator on a study funded by Guardant Health, through a contract with Kaiser Permanente Center for Health Research, to assess the adherence to a commercially available blood test for colorectal cancer. The rest of the authors declare they have no competing interests.”

The original article [1] has been updated.

## References

[1] Coury J, Coronado G, Currier JJ (2024). Methods for scaling up an outreach intervention to increase colorectal cancer screening rates in rural areas. Implement Sci Commun.

